# The Associations of Cardiometabolic and Dietary Variables with Clinical Periodontitis in Adults with and without Type 2 Diabetes: A Cross-Sectional Study

**DOI:** 10.3390/nu16010081

**Published:** 2023-12-26

**Authors:** Arpita Basu, Leigh Ann Richardson, Alicia Carlos, Neamat Hassan Abubakr, Robin L. Weltman, Jeffrey L. Ebersole

**Affiliations:** 1 Department of Kinesiology and Nutrition Sciences, School of Integrated Health Sciences, University of Nevada at Las Vegas, Las Vegas, NV 89154, USA; richal7@unlv.nevada.edu; 2 Department of Biomedical Sciences, School of Dental Medicine, University of Nevada at Las Vegas, Las Vegas, NV 89154, USA; carloa2@unlv.nevada.edu (A.C.); neamat.hassan@unlv.edu (N.H.A.); jeffrey.ebersole@unlv.edu (J.L.E.); 3 Department of Clinical Sciences, School of Dental Medicine, University of Nevada at Las Vegas, Las Vegas, NV 89154, USA; robin.weltman@unlv.edu

**Keywords:** periodontitis, vitamin D, fruit, vegetable, diabetes, C-reactive protein, bleeding score

## Abstract

Periodontitis is a commonly occurring inflammatory oral disease affecting a large proportion of global and US adults and is characterized by the destruction of the tooth-supporting apparatus. Its etiology is multifactorial, and type 2 diabetes and diet play critical roles in its remission and progression. However, few studies have addressed nutritional and serum vitamin D status in adults with periodontitis in the presence of diabetes. A cross-sectional study (*n* = 78), and a sub-set of age- and BMI-matched case–control studies (*n* = 50), were conducted to examine differences in dietary and cardiometabolic variables, and serum vitamin D in adults with periodontitis with or without diabetes. Participants provided fasting blood samples and 24-h diet recalls on at least two different days. Data on health history, body weight, height, nutritional habits, and clinical features of periodontitis were also collected. The Mann–Whitney U Test (with exact *p*-value estimation by Monte Carlo simulation) was used to examine differences by diabetes status in continuous and ordinal variables. Results revealed significantly lower serum vitamin D, and dietary intake of fruits, vegetables, dairy, vitamins A and C in adults with periodontitis with vs. without diabetes in the sub-study (all *p* < 0.05). In the overall sample, adults with diabetes presented with higher caries risk measures and lower numbers of teeth than those without diabetes; plaque and bleeding scores did not differ by diabetes status. Finally, a significant associations of food habits was observed, especially consuming protein-rich foods twice a day with a lower bleeding score, and daily consumption of fried or fast foods with a fewer number of teeth present (all *p* < 0.05). The present findings show significant dietary and serum vitamin D inadequacies among adults with periodontitis, and diabetes further aggravates the observed malnourishment and oral health.

## 1. Introduction

Periodontitis, the most common chronic inflammatory non-communicable disease affecting >40% of US adults, is characterized by a progressive destruction of the tooth-supporting apparatus [1]. Its primary features include the loss of periodontal tissue support that is manifested through clinical attachment loss (CAL) and radiographically assessed alveolar bone loss, the presence of periodontal pocketing and associated gingival bleeding [2]. According to the Global Burden of Disease Study (2019), severe periodontitis has been steadily increasing over the past three decades [3], and much of this has been attributed to coexisting conditions/behaviors, such as diabetes, smoking and tobacco use, poor lifestyle factors, and the general aging process [4,5,6,7,8]. The pathogenesis of periodontitis is initiated primarily by bacteria in the subgingival sulcus, especially anaerobic species, e.g., *Porphyromonas gingivalis*, *Treponema denticola*, and *Tannerella forsythia*, that lead to a sequelae of persistent host inflammatory responses leading to degradation of the periodontal tissues and tooth loss [9,10,11]. As expected, periodontitis contributes to poor dietary choices and nutritional status, which further sustains host malnourishment, and progression to more advanced disease. Nutritional and oral hygiene factors have also been shown to increase risk of dental caries that lead to the eventual development of periodontitis [12].

Among the prevalent related chronic conditions, diabetes mellitus, especially type 2 (T2D) has been strongly linked to periodontitis in a bi-directional manner. In a reported meta-analysis of 53 observational studies, the adjusted prevalence of T2D was approximately four-fold higher in those with periodontitis, and the prevalence of periodontitis was approximately two-fold higher in those with T2D [13]. The pathways linking periodontitis with diabetes have been mainly understood to revolve around hyperglycemia, which impairs immune function and thereby, cytokine biology, as well as the presence of periodontal inflammation leading to impaired systemic glycemic control [4]. In a systematic review and meta-analysis of nine randomized controlled trials (RCTs), nonsurgical periodontal treatment of scaling and root planing (SRP) was shown to improve glycemic control and C-reactive protein significantly, a biomarker of inflammation in adults with T2D [14].

Few studies have reported how dietary intake and nutrition status interplay with diabetes in determining the clinical severity of periodontitis. Among the essential nutrients, vitamin D has been shown to affect periodontitis, and lower serum vitamin D levels have been reported in adults with periodontitis when compared to those without the condition [15]. Studies have also shown that vitamin D supplementation with SRP improves CAL, but not other clinical indices [16]. Recent substantial evidence supports that vitamin D can regulate inflammatory/immune cells [17,18]. These anti-inflammatory impacts can modulate gingivitis, which is a precursor of periodontitis, by promoting normal mineralization of bones and teeth, as well as preventing alveolar bone loss [19]. Vitamin D has also been shown to lower insulin resistance in muscle and liver tissues, thus lowering diabetes risk [20]. A recent meta-analysis of RCTs revealed vitamin D supplementation reduced the risk of new-onset diabetes by 15% in adults with prediabetes [21]. Toothpaste containing vitamin D and other paraprobiotics have also been shown to re-duce gingival bleeding in periodontal patients [22]. More detailed nutritional studies are highly needed in adults with co-existing T2D and periodontitis.

Data on the association between habitual dietary intake and clinical periodontitis are generally limited. A cross-sectional study of the US National Health and Nutrition Examination Survey (NHANES) showed an inverse association of a dietary pattern of salad, fruits and vegetables, and beverages including water or tea, with the extent of CAL in adults with diabetes [23]. These observations corroborate our previous findings reported from an NHANES dataset, which showed lower serum carotenoid levels, indicative of fruit and vegetable intake in adults with periodontitis [24]. Dietary patterns rich in fruits and vegetables also provide sources of micronutrients, especially vitamins A, C and E that are essential for the strength of the connective structure of the soft tissues and teeth, as well as prevent oxidative stress and inflammation that underlie the pathogenesis of periodontitis [25,26,27,28]. In another NHANES analysis, a higher adherence to dietary patterns that are pro-inflammatory was associated with an increased risk of periodontitis and tooth loss in adults; adults who did not have diabetes and consumed a less pro-inflammatory diet revealed a lower risk of tooth loss [29]. Thus, diabetes itself poses a metabolic burden that requires a higher degree of adherence to a healthy diet and lifestyle to counteract the hyperglycemia-related systemic challenges including impaired oral health. However, previous reports do not address serum vitamin D levels and dietary status with detailed clinical indices of periodontitis, and how they are affected by diabetes. Thus, to further understand the interplay of diabetes, and dietary and cardiometabolic variables with clinical periodontitis, we conducted a cross-sectional study among adults with periodontitis with or without T2D. We examined differences in cardiometabolic and clinical variables in periodontitis by diabetes status, as well by serum vitamin D in these adults. We examine the null hypothesis that there are no differences in dietary intakes and serum vitamin D, as well as in clinical features of the disease in adults with periodontitis with and without diabetes.

## 2. Methods

### 2.1. Study Design

A cross-sectional study of adults with diagnosed periodontitis on maintenance therapy was conducted at the School of Dental Medicine at the University of Nevada Las Vegas (UNLV) between October 2022 and November 2023. Periodontitis was defined as having at least four teeth in two different quadrants with pocket probing depth (PPD) of ≥5 mm, CAL of ≥2 mm and bleeding upon probing (BOP) [30]. All participants provided written informed consent, and the study was approved by the ethics committee at UNLV (UNLV-2022-111). The inclusion criteria was comprised of established periodontitis on maintenance therapy involving a dental visit once in three months, clinical diagnosis of T2D based on the guidelines of the American Diabetes Association [31], and willingness to provide a serum sample and at least two 24-h dietary recalls. Exclusion criteria involved adults with no diagnosis of periodontitis, on special diets, pregnant or lactating, undergoing major treatment, such as bariatric surgery for weight loss or chemotherapy, and unable to provide consent. A total of 78 adults were selected in two groups: periodontitis with or without T2D and were referred from the UNLV dental clinic by the periodontist (RLW).

### 2.2. Data Collection and Analyses

Demographics, clinical indices of periodontitis, anthropometrics, health history, nutritional and lifestyle data were collected from each participant’s recent clinic visit. In addition, a subset of age- and BMI-matched participants (*n* = 50) was selected from the larger sample of adults with periodontitis with or without T2D. In this sub-set, each participant provided a blood sample and a detailed 24-h dietary recall for at least two to three days that was collected during the study visit. Freshly drawn blood samples were sent to Quest Diagnostics (Las Vegas, NV, USA) for analyses of serum vitamin D, glycated hemoglobin (HbA1c), and C-reactive protein (CRP). Serum vitamin D status was defined as follows: deficiency (<20 ng/mL), insufficiency (20–29 ng/mL), and optimal (≥30 ng/mL). Dietary data were analyzed using ESHA’s Food Processor^®^ Nutrition Analysis software (Version 11.7) using the household measures of foods and ingredients in self-reported dietary recalls by the participants. A trained dietetic assistant entered all data in the software and calculated the daily amounts of caloric intake, the energy contributions of carbohydrates, fats, and proteins, the amounts of major food groups consumed (fruits, vegetables, and dairy), and intake of selected essential vitamins and minerals. Nutritional behaviors associated with periodontitis were recorded. These included the regular consumption of at least five total servings of fruits and vegetables, two servings of protein foods, four servings of whole grains, less than one serving of dairy foods, daily consumption of fried/fast/oily foods, and whether one consumed snacks and sweetened beverages in between meals, as well as adequacy of water intake.

### 2.3. Statistical Analyses 

Our main objective was to examine clinical indices of periodontitis, cardiometabolic and dietary variables by diabetes status and examine their association with serum vitamin D and dietary intake. All data variables were summarized as a total sample, as well as by diabetes status among adults with periodontitis. For binary and multi-categorical variables, descriptive statistics were calculated as total *n* as well as column percentage for each category, and as median (interquartile range) for count and continuous variables. Chi-square (or Fisher’s Exact) test was performed to examine differences in binary and multi-categorical variables by diabetes status. The Mann-Whitney U Test (with exact *p*-value estimation by Monte Carlo simulation) was used to examine differences by diabetes status in continuous and ordinal variables. Spearman (and Kendall Tau b for CRA analyses) correlational analyses were also performed to examine the associations of serum vitamin D, CRP, and fruit and vegetable intake with clinical periodontitis factors. A sample of 25 in each group (periodontitis with or without diabetes) was adequate to detect a difference of 5.5 ± 2.6 ng/mL in serum vitamin D with 80% power based on a previous report [32]. Statistical analyses were performed using SAS v9.4 (SAS Institute, Cary, NC, USA). A two-sided alpha level of 0.05 was used to define statistical significance.

## 3. Results

### 3.1. Participant Characteristics and Clinical Indices of Periodontitis

Table 1 summarizes the demographics, anthropometric data, health history and clinical periodontitis features in the total patient cohort, as well as stratified by diabetes status. Observed, age, presence of hypertension, hyperlipidemia, and medication usage were significantly higher, and alcohol use was lower among those with T2D. While the stage of periodontitis, plaque score, and bleeding score did not differ by diabetes status, tooth retention, mobility, furcation, and pocket depths (≥4 mm) were significantly lower in the diabetic group (all *p* < 0.05). In the total cohort, a higher percentage of adults with diabetes showed a ‘high caries risk assessment’ measure compared to those without diabetes. Sex and ethnicity did not vary in adults with periodontitis with or without diabetes.

### 3.2. Nutritional Data, Serum Vitamin D, and CRP

As shown in Table 2, nutritional habits did not differ significantly between the two groups. However, adults with periodontitis and concomitant diabetes had a lower consumption of the recommended amounts of fruits, vegetables, whole grains and dairy than those without diabetes (Table 2). When examining the actual intake of essential food groups based on 24-h recalls (Table 3), the total intake of fruits, vegetables and dairy was significantly lower in those with diabetes in the presence of periodontitis. Concomitant intake of vitamins A and C was also significantly lower in the diabetes group. Serum vitamin D was significantly lower, and serum CRP was significantly higher in adults with periodontitis with diabetes compared to individuals without diabetes (Table 3). 

### 3.3. Correlations of Serum Vitamin D, CRP, Food Group Intake and Nutritional Behaviors with Clinical Measures of Periodontitis

As shown in Table 4, no significant correlations were observed in the sub-sample of adults with periodontitis with or without diabetes, except a significant negative correlation between CRP and bleeding score (*p* < 0.05). In Table 5, adherence to specific food group intake, such as daily intake of fried/fast/oily foods and protein-rich foods, was significantly different with the number of teeth and bleeding score, respectively (*p* < 0.05).

## 4. Discussion

This observational cross-sectional study and a sub-set of age- and BMI-matched case–control analyses of adults with or without diabetes in moderate to severe periodontitis, revealed many essential findings based on which we reject the null hypothesis. Serum vitamin D, an indicator of cardiometabolic, bone, and immune health was low in the total cohort, and significantly lower in adults with periodontitis and concomitant diabetes. On the other hand, CRP, a clinically used biomarker of inflammation was elevated in the total cohort and was significantly higher in adults with periodontitis and concomitant diabetes. Dietary intake of food groups that provide the essential micronutrients for periodontal health, such as fruits, vegetables, and dairy, as well as vitamins A and C, were significantly lower in those patients with diabetes. Correlating nutritional habits with clinical periodontitis revealed healthy dietary habits, such as consuming protein rich foods twice a day to be associated with lower bleeding score, and unhealthy dietary habits, such as daily consumption of fried and fast foods to be associated with a fewer present teeth. These observational data provide strong evidence of the role of diet quality in clinical periodontitis and potentially an increased emphasis on addressing these deficiencies in routine dental care. Furthermore, our findings add to the body of literature showing several other emerging therapies, such as using ozonated compounds in gel may reduce periodontitis in diabetes patients [33].

Previously reported cross-sectional studies in US, African, and European populations reveal inverse associations of healthy dietary patterns, especially of plant-based anti-inflammatory diets, with the prevalence of periodontitis [7,34,35]. However, while diabetes status was adjusted in the model, these studies did not report how dietary intake of food groups and nutrients, and clinical periodontitis differed by diabetes status. Furthermore, nationally representative samples do not always reflect the regional differences in diet-disease status that may be affected by variations in ethnicities and access to dental care. The current age- and BMI-matched cases of periodontitis with or without diabetes reveal that diabetes significantly impacted the dietary intake of fruits, vegetables, and dairy. The present findings conform to previous observational studies showing higher intake of these food groups related to lower incidence of type 2 diabetes, as well as progression of periodontitis [36,37,38,39,40]. Bioactive compounds in whole fruits and vegetables (especially the polyphenols and fiber) and dairy products have been shown to improve inflammatory biomarkers and alveolar bone loss in gingivitis and periodontitis [38,41,42]. On the other hand, impaired masticatory abilities [43,44] and changes in taste and smell in progressive periodontitis [45,46] may substantially limit or skew the intake of whole fruits and vegetables, as well as impact the preference of specific foods in older adults affected by periodontitis and diabetes. Fruits, vegetables, and dairy are nutrient-dense foods that are significant contributors to essential micronutrients, especially vitamins A, C and E, as well as fibers that promote periodontal and gut microbiome health [7,35,47,48,49,50]. Their intake below the recommended levels, especially in those with diabetes, may explain the elevated prevalence of caries risk, as well as lower number of teeth present when compared to those without diabetes that was observed in the present study.

Vitamin D plays a key role in optimal periodontal health by preventing alveolar bone loss, promoting normal tooth mineralization, and lowering oxidative stress and inflammation [16]. While some cross-sectional studies show lower serum vitamin D in periodontitis [32,51,52], others show no differences [53,54]. Again, many of these studies did not report and/or adjust for vitamin D supplementation within a recent timeframe that may mask the basal levels in triggering risks of chronic periodontitis. Using a cross-sectional sample of US adults, we have previously reported serum vitamin D to be significantly lower among those with periodontitis, as well as lower levels with increasing periodontitis severity [24]. Few reported studies have compared serum vitamin D between diabetes and no diabetes in the context of periodontitis. In a case–control study conducted in Iran among adults, periodontitis plus T2D, and no periodontitis, revealed serum vitamin D in the average range of 14 to 17 ng/mL with no significant differences among the three groups [53]. On the other hand, a cross-sectional study in Chinese adults revealed significantly lower serum vitamin D in patients with periodontitis plus T2D versus those without diabetes. Patients with periodontitis with or without T2D had a mean serum vitamin D level of approximately 19 ± 3 and 24 ± 3 ng/mL, respectively [55]. This observational study also revealed lower vitamin D receptor expression in the gingival tissues of adults with periodontitis with diabetes; the researchers explained this as an outcome of low serum vitamin D and elevated inflammatory status, specifically via downregulation of the anti-inflammatory protein tyrosine phosphatase non-receptor type 2 gene (PTPN2) in diabetes [55]. A similar significant association of the combined effect of vitamin D insufficiency and periodontitis, with presence of diabetes, but not prediabetes, was also reported by another cross-sectional study in US adults [56]. The current case–control study also provided evidence on the depletion effects of diabetes on serum vitamin D, which have been explained by several mechanisms. These include the increased oxidative stress and inflammatory burden of chronic hyperglycemia, low dietary intake of vitamin D-specific foods and sunlight exposure, and reduced expressions of vitamin D receptors and anti-inflammatory genes [57]. Overall dairy intake in the present study adults with periodontitis did not meet the minimum USDA-based dietary recommendation for this food group, and intake was even lower in those with diabetes. Dairy products, especially milk, cheese and yogurt are fortified with vitamin D and are naturally rich in bioavailable calcium and phosphorus essential for teeth and bone health [15,57]. Thus, dietary consultations focusing on these essential food groups should be emphasized as part of routine dental care and oral health wellness. Overall, results showed no significant correlation between vitamin D serum and the individual clinical variables of periodontitis progression, which could be explained by the overall small sample size and the single time point determination of serum vitamin D in the current study. Thus, the association of circulating vitamin D with periodontitis severity and the modifying role of diabetes deserve further investigation in longitudinal studies of these diseases in adults.

Dietary habits, such as twice daily consumption of protein-rich foods, revealed lower median bleeding score when compared to those who did not consume this amount. Protein-rich foods, such as meat, fish, eggs, beans, and nuts provide a range of nutrients in addition to essential amino acids, such as omega-3-fatty acids, zinc and iron that maintain normal hemostatic factors and promote wound healing [58,59,60]. In a cross sectional study of adults, periodontal healing after SRP was significantly higher in those who consumed the recommended levels of dietary protein, compared to those with an inadequate intake in a non-smoking populations [61]. On the other hand, it was also observed unhealthy dietary habits, such as daily consumption of fried/fast/oily foods were associated with fewer present teeth. The possible explanation for this observation is that these types of foods are high in total fats and calories, and typically low in essential nutrients, such as antioxidant vitamins, minerals, and fiber, thereby leading to malnourishment precipitating suboptimal periodontal health. In a systematic review and meta-analysis of 27 studies, increasing consumption of the category of ultra-processed foods (e.g., fried, ready-to-eat, other processed foods) showed a positive association with dental caries in children and adolescents [62], as well as with moderate/severe periodontitis in adults [63]. Limitations of the current study included the lack of a comparison group of adults with no periodontitis without diabetes. This cross-sectional analysis, as well as the case–control sub-study, does not address causality. We did not quantify biomarkers of fruit and vegetable intake, such as serum carotenoids and antioxidant status. Finally, we did not examine social and behavioral factors beyond diet that may affect these conditions.

In conclusion, our observational study clearly shows significant associations in a well-defined adult population with periodontitis and on maintenance therapy, thereby providing useful data on the role of serum vitamin D and nutritional status that differ by diabetes status. This should be considered by dental care providers in assessing periodontal health and risk for periodontitis and its progression in future longitudinal studies. Thus, a more detailed screening for fundamental patient dietary habits and vitamin D status and providing targeted nutritional advice would appear to provide a positive benefit in prevention and routine maintenance care of patients with periodontitis.

## Figures and Tables

**Table 1:** Demographics and clinical indices of periodontitis.

	Total Cohort (*n* = 78)	Non-DM (*n* = 40)	DM (*n* = 38)	*p*-Value (Chi-Square)
Variables	*n* (%)			
Gender				0.6778
Male	47 (60)	25 (63)	22 (58)	
Female	31 (40)	15 (38)	16 (42)	
Age				**0.0053 ^#^**
<55	17 (22)	14 (35)	3 (8)	
≥55	61 (78)	26 (65)	35 (92)	
Ethnicity				0.4677 ^#^
African/AA	23 (29)	9 (23)	14 (37)	
Caucasian	32 (41)	17 (43)	15 (39)	
Hispanic	17 (22)	11 (28)	6 (16)	
Multiple/Other	6 (8)	3 (8)	3 (8)	
BMI				0.1331 ^#^
18.5–24.9	9 (12)	5 (13)	4 (11)	
25–29.94	27 (35)	18 (45)	9 (24)	
>30	34 (44)	14 (35)	20 (53)	
Diabetes				--
Yes	38 (49)	--	--	
No	40 (51)	--	--	
Hypertension				**0.0001**
Yes	42 (54)	13 (33)	29 (76)	
No	36 (46)	27 (68)	9 (24)	
Hyperlipidemia				**0.0016**
Yes	37 (47)	12 (30)	25 (66)	
No	41 (53)	28 (70)	13 (34)	
Medication				**<0.0001 ^#^**
None	12 (15)	12 (30)	0 (0)	
OTC	3 (4)	3 (8)	0 (0)	
Rx-only	38 (49)	17 (43)	21 (55)	
Both (OTC and Rx)	25 (32)	8 (20)	17 (45)	
Alcohol Use				**0.0158**
Yes	35 (45)	23 (58)	12 (32)	
No	42 (54)	16 (40)	26 (68)	
Social History				0.2863 ^#^
No Tobacco/Ethanol Use	64 (82)	31 (78)	33 (87)	
Tobacco/Nicotine only	7 (9)	3 (8)	4 (11)	
Marijuana	6 (8)	5 (13)	1 (3)	
Tobacco/Nicotine + Marijuana	1 (1)	1 (3)	0 (0)	
Tobacco Use				0.6402
Yes	7 (9)	3 (8)	4 (11)	
No	71 (91)	37 (92)	34 (89)	
CRA				**0.0003 ^#^**
High	31 (40)	10 (25)	21 (55)	
Moderate	32 (41)	25 (68)	7 (18)	
Low	7 (9)	2 (13)	5 (13)	
Periodontal Status				0.3994 ^#^
Stage 2	19 (24)	8 (20)	11 (29)	
Stage 3	50 (64)	27 (68)	23 (61)	
Stage 4	7 (9)	5 (13)	2 (5)	
Plaque Score				0.9552
1–20%	16 (21)	9 (23)	7 (18)	
21–40%	20 (26)	10 (25)	10 (26)	
41–60%	12 (15)	7 (18)	5 (13)	
61–80%	17 (22)	8 (20)	9 (24)	
81–100%	13 (17)	6 (15)	7 (18)	
Bleed Score				0.1897 ^#^
1–20%	34 (44)	13 (33)	21 (55)	
21–40%	19 (24)	12 (30)	7 (18)	
41–60%	9 (12)	5 (13)	4 (11)	
61–80%	13 (17)	7 (18)	6 (16)	
81–100%	3 (4)	3 (8)	0 (0)	
Arch Pocket Depth, Deepest				0.5151
Maxillary	40 (51)	22 (55)	18 (47)	
Mandibular	22 (28)	9 (23)	13 (34)	
Both	16 (21)	9 (23)	7 (18)	
Dentition Pocket Depth, Deepest *				
Molar	63 (81)	35 (88)	28 (74)	0.1217
Premolar	14 (18)	2 (5)	12 (32)	**0.0028 ^#^**
Canine	8 (10)	2 (5)	6 (16)	0.1489 ^#^
Lateral	5 (6)	2 (5)	3 (8)	0.6710 ^#^
Central	6 (8)	3 (8)	3 (8)	1.0000 ^#^
	Median (IQR) ^§^			
Number of Teeth Present	25 (21–27)	26 (23–27)	24 (17–27)	**0.0299**
Number of Teeth with Mobility	3 (1–5)	3 (2–8)	2 (1–4)	0.0641
Number of Teeth with furcation involvement	5 (2–8)	8 (4–11)	3 (1–5)	**<0.0001**
Number of Pocket Depths ≥4 mm	29 (17–47)	35 (21–58)	20 (11–34)	**0.0048**
Deepest Pocket Depth	6 (5–8)	8 (6–9)	6 (5–8)	**0.0015**

* Not mutually exclusive groups. Some individuals had similar depths across multiple dentition pocket locations. ^§^ *p*-values from Monte Carlo Estimates for the Exact Test. ^#^ Fisher’s Exact Test. *p* < 0.05 in bold font. Abbreviations: AA: African American; CRA: caries risk assessment; DM: diabetes mellitus; IQR: interquartile range; OTC: over the counter; Rx: prescription.

**Table 2:** Self-reported dietary habits in adults with periodontitis with and without diabetes.

	Total Cohort (*n* = 78)	No Diabetes (*n* = 40)	Presence of Diabetes (*n* = 38)	*p*-Value (Chi-Square)
Variables	*n* (%)	*n* (%)	*n* (%)	
Eat/drink ≥5 times a day? (Yes)	39 (50)	23 (58)	16 (42)	0.3734
Do you chew regular (non-sugar free) gum? (Yes)	11 (14)	7 (18)	4 (11)	0.5328 ^#^
Sweetened beverages between meals/in place of meals? (Yes)	24 (31)	15 (38)	9 (24)	0.3156
Snacks between meals or in place of meals? (Yes)	35 (45)	19 (48)	16 (42)	0.9843
Consume dairy <1x/day? (Yes)	39 (50)	23 (58)	16 (42)	0.3734
Fried/fast/oily foods daily? (Yes)	17 (22)	7 (18)	10 (26)	0.2124
Whole grain breads/cereals at least 4x/day? (Yes)	18 (23)	11 (28)	7 (18)	0.5000
Fruit/vegetables at least 5x/day? (Yes)	26 (33)	14 (35)	12 (32)	0.9547
Meat, poultry, fish, eggs, beans, nuts 2x/day? (Yes)	46 (59)	27 (68)	19 (50)	0.3052
6–8 cups of water daily? (Yes)	58 (74)	31 (78)	27 (71)	0.7572
Average daily alcohol intake?				0.4010
<1	56 (72)	29 (73)	27 (71)	
>1	14 (18)	9 (23)	5 (13)	

^#^ *p*-value from Fisher’s exact test.

**Table 3:** Nutritional intakes, serum C-reactive protein and vitamin D among age and BMI-matched adults with periodontitis by diabetes status.

	Total Cohort (*n* = 50)	No Diabetes (*n* = 25)	Presence of Diabetes (*n* = 25)	*p*-Value ^§^
Gender: Female (*n*, %)	22 (44)	12 (48)	10 (40)	0.5804
Variables	Median (IQR)			
Age	62 (57–66)	59 (53–66)	65 (58–66)	0.2442
BMI	29 (28–32)	29 (28–31)	31 (28–32)	0.2081
Serum Vitamin D, ng/mL	24.0 (18–31)	31 (24–37)	21 (17–24)	**0.0002**
C-Reactive Protein, g/L	4.6 (3.7–5.5)	3.8 (2.8–4.6)	5.3 (4.4–6.4)	**0.0001**
HbA1c, %	6.0 (4.9–7.1)	4.9 (4.7–5.2)	7.1 (6.8–7.3)	**<0.0001**
Calories, kcal/day	1998 (1867–2134)	1986 (1890–2111)	2113 (1867–2143)	0.2116
Carbohydrates, %kcal/day	60 (55–65)	56 (55–62)	61 (58–65)	**0.0259**
Fats, %kcal/day	18 (15–21)	17 (15–20)	20 (18–22)	**0.0041**
Proteins, %kcal/day	23 (17–28)	25 (23–30)	18 (16–23)	**0.0003**
Fruits, cups/day	0.3 (0.2–0.5)	0.4 (0.3–0.6)	0.2 (0.2–0.4)	**0.0036**
Vegetables, cups/day	0.5 (0.3–0.8)	0.5 (0.5–0.8)	0.3 (0.2–0.7)	**0.0037**
Dairy, cups/day	0.8 (0.5–1.1)	1.1 (0.8–1.2)	0.5 (0.3–1.0)	**<0.0001**
Vitamin A, µg/day	387 (309–458)	431 (312–489)	342 (287–421)	**0.0222**
Vitamin C, mg/day	27 (22–38)	37 (28–44)	25 (21–27)	**<0.0001**
Vitamin E, mg/day	9.3 (7.2–11.0)	8.8 (7.3–11)	10 (6–11)	0.6842
Iron, mg/day	7.1 (5.7–8.3)	7.2 (6.4–8.1)	7 (5.1–8.5)	0.5599
Zinc, mg/day	4.5 (3.3–6.2)	5.3 (3.6–6.3)	4.2 (2.6–6.2)	0.1314
Fluoride, mg/day	1.1 (0.8–1.3)	1.1 (0.8–1.5)	1.1 (0.8–1.2)	0.6496

^§^ *p*-values from Monte Carlo Estimates for the Exact Test. *p* < 0.05 in bold font.

**Table 4:** Spearman correlations for risk variables with clinical periodontitis.

Variables	Associated Variables	Correlation Coefficient	*p*-Value
Serum Vitamin D	CRA ^#^	r = 0.00	0.9834
	Plaque Score	r = −0.09	0.5485
	Bleed Score	r = 0.14	0.3317
	No. of Teeth	r = −0.19	0.1761
Fruit and Vegetable Intake	CRA ^#^	r = 0.19	0.2092
	Plaque Score	r = −0.20	0.1598
	Bleed Score	r = 0.03	0.8461
	No. of Teeth	r = 0.10	0.4784
Serum C-Reactive Protein	CRA ^#^	r = −0.09	0.5485
	Plaque Score	r = −0.15	0.3110
	Bleed Score	r = −0.31	**0.0286**
	No. of Teeth	r = 0.04	0.7760

^#^ Kendall Tau b correlations were also conducted with no significant changes in correlation coefficients or *p*-values. *p* < 0.05 in bold font. Abbreviation: CRA: caries risk assessment.

**Table 5:** Comparison of nutrition behaviors and outcomes: CRA, plaque score, bleed score, and number of teeth ^§^.

Nutrition Question and Responses		*n*	CRA *			Plaque Score			Bleed Score			No. of Teeth		
			Median Status	Range	*p*-Value	Median Range	IQR	*p*-Value	Median Range	IQR	*p*-Value	Median	IQR	*p*-Value
Sweetened beverages between meals/in place of meals?	Yes	24	Moderate	High -Moderate	0.7492	41–60%	(21–80%)	0.5107	21–40%	(1–60%)	0.8087	26	(20–28)	0.7088
	No	48	Moderate	High-Moderate		41–60%	(21–80%)		21–40%	(1–80%)		25	(22–27)	
Consume dairy <1x/day?	Yes	39	Moderate	High-Moderate	0.5845	41–60%	(21–80%)	0.9677	21–40%	(1–80%)	0.2127	25	(20–27)	0.9493
	No	33	High	High-Moderate		41–60%	(21–80%)		1–20%	(1–80%)		26	(22–27)	
Fried/fast/oily foods daily?	Yes	17	High	High-Moderate	0.6280	41–60%	(21–80%)	0.6932	21–40%	(1–40%)	0.3721	21	(16–24)	**0.0179**
	No	53	Moderate	High-Moderate		41–60%	(21–80%)		21–40%	(1–80%)		26	(23–27)	
Whole grain breads/cereals at least 4x/day?	Yes	18	Moderate	High-Moderate	0.8635	61–80%	(21–80%)	0.3173	21–40%	(21–80%)	0.7410	26	(22–28)	0.4945
	No	52	Moderate	High-Moderate		41–60%	(21–80%)		21–40%	(1–80%)		25	(21–27)	
Fruit/vegetables at least 5x/day?	Yes	26	Moderate	High-Moderate	0.8387	61–80%	(41–80%)	0.0627	21–40%	(1–60%)	0.8265	26	(22–28)	0.7212
	No	44	Moderate	High-Moderate		41–60%	(21–80%)		21–40%	(1–80%)		25	(21–27)	
Meat, poultry, fish, eggs, beans, nuts 2x/day?	Yes	46	Moderate	High-Moderate	0.2482	41–60%	(21–80%)	0.2914	21–40%	(1–60%)	**0.0474**	26	(22–28)	0.4615
	No	24	High	High-Moderate		61–80%	(21–100%)		41–60%	(1–80%)		25	(21–27)	

^§^ *p*-values from Monte Carlo Estimates for the Exact Test; *p* < 0.05 in bold font. * *n* values differed for CRA for the following questions: Consume dairy <1x/day? Yes: 37|No: 33; Fried/fast/oily foods daily? Yes: 17|No: 51; Whole grain breads/cereals at least 4x/day? Yes: 17|No: 51; Fruit/vegetables at least 5x/day? Yes: 25|No: 43; Meat, poultry, fish, eggs, beans, nuts 2x/day? Yes: 44|No: 24. Abbreviation: CRA: caries risk assessment.

## Data Availability

The data are not publicly available due to patient privacy.
